# A European algorithm and strategy for management of chronic postoperative inguinal pain (EurA-CPIP)

**DOI:** 10.3389/jaws.2026.16944

**Published:** 2026-10-14

**Authors:** K. G. Andersen, T. W. K. van der Meer, N. van Veenendaal, N. B. Foss, M. J. A. Loos, M. Miserez, M. Pawlak, R. M. H. Roumen, T. Naddaf, M. Togsverd-Bo, M. W. Stadeager, E. K. Aasvang, W. A. R. Zwaans

**Affiliations:** 1 Department of Anaesthesia and Intensive Care, Hvidovre Hospital, University of Copenhagen, Copenhagen, Denmark; 2 Department of Clinical Medicine, Copenhagen University, Copenhagen, Denmark; 3 SolviMáx Center of Expertise for Complex Groin and Abdominal Wall Pathology, Eindhoven, Netherlands; 4 Department of General Surgery, Máxima Medical Center, Eindhoven, Netherlands; 5 Department of Anaesthesiology, University Medical Center Utrecht, Utrecht, Netherlands; 6 Department of Abdominal Surgery, University Hospitals Leuven, Leuven, Belgium; 7 Department of General Surgery, Golden Jubilee University National Hospital, Glasgow, United Kingdom; 8 School of Medicine, Dentistry & Nursing, University of Glasgow, Glasgow, United Kingdom; 9 Gastrounit, Surgical Section, Centre for Surgical Research, Hvidovre Hospital, University of Copenhagen, Copenhagen, Denmark; 10 Department of Anaesthesiology, Center for Cancer and Organ Diseases, Rigshospitalet, Copenhagen, Denmark; 11 NUTRIM School of Nutrition and Translational Research in Metabolism, Maastricht University Medical Center, Maastricht, Netherlands

**Keywords:** chronic postoperative inguinal pain (CPIP), inguinal hernia repair complications, persistent postsurgical pain, postherniorrhaphy pain, treatment algorithm, multidisciplinary care

## Abstract

**Background:**

Chronic postoperative inguinal pain (CPIP) is a disabling complication affecting 8%–17% of all patients after inguinal hernia repair. It is a complex pain condition with several pathophysiological mechanisms involved, including neuropathic pain, inflammation, and mechanical effects (e.g., mesh or scar tissue). Management remains inconsistent, probably due to limited high-quality evidence, heterogeneous approach, and insufficient standardisation of outcomes. This study aimed to develop EurA-CPIP, a multidisciplinary treatment algorithm and prospective data-collection platform designed to support consistent phenotyping, step-up management, and standardised outcomes across centres, creating an iterative platform for future hypothesis-driven research and multicentre trials.

**Methods:**

An international multidisciplinary working group from European tertiary referral centres developed the European Algorithm and Strategy for Management of Chronic Postoperative Inguinal Pain (EurA-CPIP). Development was informed by the evidence synthesis underpinning the updated international HerniaSurge guidelines and a critical appraisal of previously published CPIP algorithms. A pragmatic step-up algorithm was designed, integrating pharmacological, interventional, surgical and supportive treatments, with prospective outcome registration and planned data-driven revision on a regular basis.

**Results:**

EurA-CPIP provides a multidisciplinary algorithm with entry at multiple levels depending on prior treatment attempts. The concept is to begin and, if needed, advance from conservative measures such as systemic medication and topical therapy, to more invasive treatments such as trigger point injections, and ultrasound-guided nerve blocks; predefined investigational add-ons (radiofrequency, cryoneurolysis, botulinum toxin A) are embedded after documented block response. Multidisciplinary team review precedes escalation to more invasive interventions such as surgery. A supportive and rehabilitative coping trajectory is integrated early on. Standardised outcomes include patient-reported pain outcomes, functional impairment, psychological factors, analgesic use, and patient global impression of improvement.

**Conclusion:**

EurA-CPIP is a pragmatic consensus-based strategy intended to harmonise and improve specialised CPIP care while utilising existing and developing emerging therapies. Future research should investigate pathophysiology and intervention effects within this step-up algorithm.

## Introduction

Chronic postoperative inguinal pain (CPIP) is a frequent complication after inguinal hernia repair, CPIP is defined as postoperative inguinal pain or discomfort rated by the patient as at least moderate, affecting daily activities, and persisting for more than 3 months [1]. A recent systematic review and meta-analysis reported prevalences of function-limiting CPIP of approximately 8% [2] to 17% [3]. Several patient and procedure-related risk factors have been described [2]. CPIP represents a major source of long-term morbidity with clinically relevant limitations in physical function and daily activities [4]. CPIP is also associated with reduced health-related quality of life [5], reduced patient satisfaction, and potentially negative socioeconomic consequences, although very sparsely investigated [6]. Psychological functioning may be compromised in a substantial subgroup, with a high prevalence of coincident psychological disorders reported in patients referred with CPIP, further supporting a biopsychosocial and multidisciplinary approach to assessment and management [7].

Despite the high global volume of groin hernia surgery, high-quality evidence guiding CPIP treatment remains limited. The HerniaSurge international guideline and its most recent update emphasise that the evidence base for many CPIP interventions is sparse and heterogeneous, with few advances over the last decade, contributing to largely weak recommendations [1, 8]. Over the past 2 decades, several algorithms for managing CPIP have been proposed [5, 9–19]. However, none has gained universal acceptance to date. A significant concern is that most existing algorithms lack a strong evidence base, standardised patient evaluation and sufficient follow-up, and primarily adopt a surgical perspective. Furthermore, the current algorithms were mostly developed without multidisciplinary input, despite CPIP patients presenting to both surgeons and pain specialists [1]. Against this background, the present work translates the broad recommendations of the updated HerniaSurge guideline into a pragmatic European clinical framework for specialised CPIP care. Whereas HerniaSurge provides the international guideline foundation, the European Algorithm and Strategy for Management of Chronic Postoperative Inguinal Pain (EurA-CPIP) is intended as a multidisciplinary implementation and prospective data-collection platform. EurA-CPIP was designed to support consistent phenotyping, step-up management, and standardised outcome capture across centres, thereby creating an iterative platform for future hypothesis-driven research and multicentre trials.

## Materials and methods

### Evidence base and appraisal

The development of EurA-CPIP was grounded in the evidence synthesis performed for the HerniaSurge guideline update on groin hernia management in adults [1]. A scoping review, rather than a systematic review, was undertaken to map all previously published CPIP algorithms. This approach was chosen to avoid duplicating the HerniaSurge guideline update and instead build upon it. For each algorithm, the underlying evidence, patient selection criteria, reported outcomes, and follow-up duration were critically appraised. Strengths and limitations were discussed within the working group and synthesised with findings from HerniaSurge update [1].

### Consensus process and governance

Five European tertiary referral centres with established expertise in CPIP management participated in the working group. Each centre contributed at least one delegate involved in the HerniaSurge CPIP guideline revision. Algorithm development proceeded through iterative consensus meetings, during which diagnostic pathways, treatment sequencing, and outcome measures were discussed and refined. Agreement was reached through structured discussion rather than formal Delphi methodology.

EurA-CPIP is explicitly designed as a dynamic algorithm, subject to regular revision based on accumulated prospective data and updated literature review. Decisions regarding revisions are made by the EurA-CPIP working group.

EurA-CPIP is intended as an expandable collaborative platform. Additional centres may join, provided that they commit to the core assessment dataset, predefined follow-up schedule, local governance and ethical approval, and prospective data sharing according to applicable legislation. Participating centres should either provide the mandatory core treatment steps locally or have access to them through formal referral pathways. Selected investigational interventions may remain centralised to high-volume centres.

The main purpose of EurA-CPIP is to systematically collect data from treatments in a structured, standardised clinical setting, to allow for the identification of patient characteristics and their relation to treatment responses that, over time, can stratify patients towards treatments with a higher probability of success.

### Algorithm design principles

It was by consensus decided that the algorithm should follow a step-up approach, progressing from least invasive to more invasive interventions, ultimately reoperation with neurectomy and/or mesh removal. This strategy aims to balance between maximum clinical benefit while minimising treatment-related morbidity. In this first version, patients may enter the algorithm at different stages, reflecting prior treatments received before referral, thereby reducing selection bias. All core treatments are available across participating centres, while selected investigational interventions may be centralised. In time, it is expected that data will allow for identifying responders/non-responders to specific treatments, thus advocating for more advanced treatments as a start, but for now, the evidence does not support this, i.e., reoperation without attempting less invasive techniques.

The EurA-CPIP Outcome assessments were selected after extensive discussion within the working group and are performed at baseline and predefined follow-up intervals (typically every 6 months), as illustrated in Figure 1.

**FIGURE 1:**
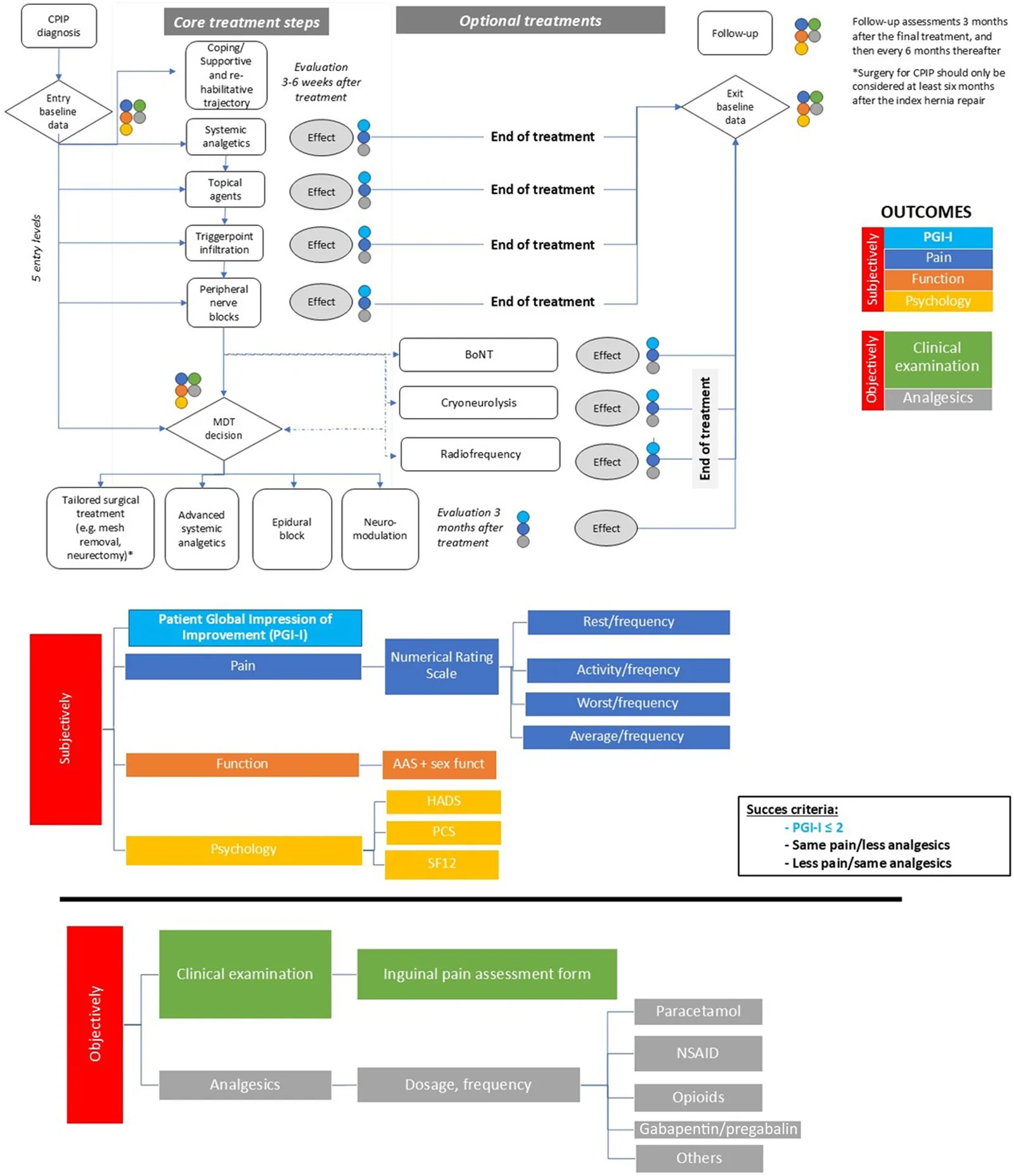
The EurA-CPIP algorithm and outcome framework. Step-up algorithm for multidisciplinary management of chronic postoperative inguinal pain. Standardised outcome framework with baseline and follow-up assessments. CPIP, chronic postoperative inguinal pain; MDT, multidisciplinary team; BoNT-A, botulinum toxin type A; PGI-I, Patient Global Impression of Improvement.

Outcomes are designed to capture patient-reported outcomes (PROMs) to quantify pain severity, functional impact, quality of life, pain phenotype (neuropathic, nociceptive/inflammatory, mechanical, nociplastic, or mixed), and key psychological modifiers relevant to CPIP at the various treatment stages to quantify effect. Instruments were selected by consensus within the working group and reviewed by an international patient panel, prioritising measures that are widely used, validated, and feasible for routine clinical implementation across centres. Where established thresholds exist, pre-specified cut-offs are applied to support standardised phenotyping and to enable consistent stratification and comparative analyses across sites and over time. Table 1 summarises the PROMs included in the EurA-CPIP dataset, the corresponding pre-defined cut-offs/responder definitions used in the outcome framework, and includes validation references for each instrument. EurA-CPIP was deliberately designed as a pragmatic clinical algorithm. Advanced pathophysiological assessments such as extended quantitative sensory testing, neurophysiology, imaging biomarkers, or other mechanistic investigations may be added within specific research protocols, but are not part of the mandatory core dataset. This was intended to preserve feasibility, reproducibility, and comparability across participating centres.

**TABLE 1:** Patient-Reported Outcome Measures included in the EurA-CPIP framework.

Instrument	Domain	Type	Pre-defined cut-off/responder definition	Validation references
NRS (numeric rating scale)	Pain severity	Primary	0–10 scale; ≥2 point reduction = clinically meaningful improvement	[20] chosen over VAS due to lower risk of bias [21]
PGI-I (patient global impression of improvement)	Perception of improvement	Primary	≤2: ‘Much’ or ‘very much’ better	​
AAS (activities assessment scale)	Functional status	Secondary	Score range, higher equals greater limitation	[22]
Sexual function questioning	Sexual function	Secondary	Presence/absence and severity of sexual dysfunction	​
HADS (hospital anxiety and depression scale)	Psychological status (anxiety and depression)	Secondary	≥8 = possible anxiety/depression; ≥11 = probable anxiety/depression	[23]
PCS (pain catastrophizing scale)	Pain catastrophising	Secondary	Total score ≥30 = clinically relevant catastrophising	[24]
SF-12 (short Form-12)	Quality of life	Secondary	PCS/MCS scores <50 = below average	[25]

### Data infrastructure and ethics

Patients are asked for consent to store and use data for research purposes, including data sharing with other centres and linkage to potential nationwide registries (health economics, genetics etc.). Each centre obtains local ethical approval in accordance with national regulations. No directly identifiable patient data are stored centrally. Participating investigators retain responsibility for maintaining the link between local records and registry case numbers. Data sharing always involves one member from each contributing centre, ensuring adherence to legislation.

## Results

### Prevention

Although EurA-CPIP primarily addresses established chronic postoperative inguinal pain, the working group recognises prevention as an integral component of the overall CPIP care pathway. Established preventive considerations include appropriate surgical indication and patient counselling, choice of operative approach, awareness and appropriate handling of the inguinal nerves, mesh and fixation strategies, surgical expertise, and recognition and management of severe postoperative pain [1, 7, 8, 26]. These measures are addressed in international guidelines and recent literature and are therefore not reproduced in detail within the present management algorithm. EurA-CPIP begins when CPIP requires structured assessment, phenotyping and treatment but preventive measures should be viewed as complementary.

### EurA-CPIP algorithm: rationale and structure

The EurA-CPIP algorithm comprises eight sequential steps (Figure 1). Progression between steps is guided by clinical response. See Supplementary Table S1 for a summary of the evidence supporting each step.

#### Step 0: Initial medical history and examination

At inclusion, the initial objective is to identify or exclude potentially treatable anatomical causes of pain and relevant competing diagnoses. This includes recurrent hernia, a meshoma or other mesh-related structural complications related to the index repair. Clinical examination and review of previous operative reports should be supplemented by imaging when a structural cause is suspected. A medical history including patient and treatment variables is collected through the standardised inguinal pain assessment form developed by the Eura-CPIP working group (Supplementary Appendix 1), including the Douleur Neuropathique 4 (DN4) and other questionnaires. Variables collected include pain debut, intensity, consequence, treatment attempts and results, comorbidities and current medications. PROMs have been filled out, and all previous groin operation reports, pain treatment reports and any radiological or other examinations must have been sent by the referring physician. The collected information supports a structured pathophysiological assessment of whether the patient has predominantly neuropathic, nociceptive/inflammatory, visceral, mechanical, nociplastic, or mixed pain features. Of note, discrimination between these types of CPIP is not always clinically possible [1].

#### Step 1: Conservative systemic pharmacological treatment

Initial management includes non-opioid systemic analgesics (paracetamol and/or NSAIDs). In patients with suspected neuropathic pain features, first-line neuropathic agents such as tricyclic antidepressants, serotonin–noradrenaline reuptake inhibitors, or gabapentinoids may be introduced. Long-term use of strong opioids is discouraged, particularly in neuropathic CPIP [27]. Treatment response is assessed after 3–6 weeks, depending on the treatment modality, dose titration, tolerability, and clinical urgency.

#### Step 2: Topical pharmacological therapies

Topical lidocaine or capsaicin patches or cream may be considered despite limited evidence of efficacy in CPIP patients. Randomised trials have not demonstrated a clear benefit, but use is not contraindicated given the low risk profile [1, 28].

#### Step 3: Trigger point injections

Trigger point injections (TPI) with local anaesthetics (e.g., lidocaine, bupivacaine, or ropivacaine), especially after Lichtenstein repair [1], are recommended as a minimally invasive diagnostic and therapeutic intervention [29–32]. Temporary pain relief following TPI supports a peripheral pain generator. No evidence-based recommendations can be given on whether TPI should be performed free-hand (‘blindly’) or ultrasound-guided [1].

#### Step 4: Peripheral nerve blocks and invasive pain treatment

Ultrasound-guided peripheral nerve blocks targeting the ilioinguinal (IIN), iliohypogastric (IHN) and/or the genital branch of the genitofemoral nerve (GBGFN) are used primarily as diagnostic (phenotyping) blocks. A single injection of low-dose local anaesthetic is performed to (i) confirm a peripheral neuropathic pain generator, (ii) map the sensory territory, and (iii) provide short-term prognostic information for subsequent targeted interventions. Evidence supporting their role in CPIP includes a placebo-controlled crossover study demonstrating analgesic/sensory effects of ultrasound-guided IIN/IHN blocks [31] and an outcomes study in CPIP comparing ultrasound guidance with nerve stimulator guidance [32].

Add-on interventions may be considered in carefully selected patients with a clinically meaningful, reproducible temporary response to diagnostic blocks. These techniques remain investigational in CPIP and should only be offered with systematic outcome registration and clear stopping rules.

##### Step 4a: Radiofrequency techniques

Pulsed radiofrequency (PRF) is a minimally neurodestructive neuromodulatory technique delivered via a radiofrequency cannula positioned adjacent to the suspected pain-generating nerve. PRF is applied in short bursts (commonly with a target tip temperature around 42 °C) with the intention of modulating nociceptive transmission without creating a conventional thermal neuroablative lesion. The clinical evidence for PRF in CPIP remains limited, and available data are derived from small, selected cohorts with heterogeneous methods and outcomes. Accordingly, PRF should be considered investigational in CPIP and, if used, implemented within a structured pathway with systematic outcome registration [33].

##### Step 4b: Cryoneurolysis

Cryoneurolysis is a percutaneous, image-guided technique in which a cryoprobe freezing at minus 20 to minus 100 °C is positioned adjacent to the suspected pain-generating nerve, causing a temporary axonotmesis with Wallerian degeneration, while preserving the connective tissue scaffold, allowing subsequent axonal regeneration and analgesia that may last weeks to months [34]. Early work in groin hernia surgery produced mixed results [35–37]. More recently, advances in ultrasound guidance and percutaneous probes have enabled more precise nerve targeting and controlled “ice-ball” monitoring, which has renewed interest in cryoneurolysis. Contemporary evidence from other surgical settings suggests that ultrasound-guided percutaneous cryoneurolysis can provide prolonged postoperative analgesia and may reduce pain for multiple weeks [38, 39]. However, CPIP-specific evidence remains limited and largely consists of small series/case reports. Cryoneurolysis should thus be considered investigational in CPIP and used only within a structured pathway with systematic outcome registration as a minimum.

##### Step 4c: Botulinum toxin A

Botulinum toxin type A (BoNT-A) is increasingly explored in peripheral neuropathic pain [40–42]. Beyond its neuromuscular effects, proposed antinociceptive mechanisms include inhibition of pain-related neurotransmitter/neuropeptide release from peripheral sensory terminals (e.g., substance P, CGRP, glutamate) with downstream attenuation of neurogenic inflammation and peripheral sensitisation; central effects via axonal transport have been hypothesised but remain debated [43, 44]. Evidence for BoNT-A in neuropathic pain is heterogeneous across indications, but contemporary reviews and meta-analyses in other focal neuropathic syndromes (e.g., postherpetic neuralgia) suggest clinically meaningful pain reduction with acceptable safety, supporting interest in more targeted, image-guided delivery approaches [45, 46]. Injections with BoNT-A adjacent to the spermatic cord have been suggested for (refractory) genitofemoral neuralgia (scrotal pain) in case series previously [47, 48]. Evidence remains very limited but demonstrates temporary but clinically relevant symptom improvement [47]. Accordingly, BoNT-A should be considered investigational in CPIP and, if used, applied within a structured pathway with systematic outcome registration and predefined criteria for continuation or discontinuation.

#### Step 5: Multidisciplinary team review

Formal MDT review is required before escalation to surgery or advanced interventional treatment. However, MDT discussion or pain-specialist assessment may occur earlier whenever phenotyping is uncertain, symptoms are severe or disabling, psychosocial complexity is substantial, or previous care has been prolonged or fragmented. Patients with persistent pain despite conservative and interventional measures must undergo multidisciplinary evaluation. Psychometric scores such as the HADS and PCS may identify high risk patients. Patients scoring ≥8 on either the HADS subscale, indicating possible depression/anxiety, or ≥11 for probable depression/anxiety [23] or PCS scores ≥30 [24], are at increased risk of poor outcomes and may benefit from early referral to a multidisciplinary team (MDT). The MDT for CPIP should at least involve an experienced hernia surgeon and a pain specialist. Ideally, the MDT should also have access to pain-specialist physiotherapy, psychological or pain-management support (see below: “supportive and rehabilitative treatments”), and relevant cross-specialty consultation such as urology, gynaecology, orthopaedics, radiology, or rehabilitation medicine, depending on the clinical presentation. The purpose of the MDT review is to refine phenotyping, exclude relevant competing diagnoses, assess psychosocial complexity, and determine whether escalation should be directed towards surgery, further interventional pain treatment, advanced pharmacological management, or supportive rehabilitation. Decisions regarding escalation are individualised. The relevance of an organised MDT structure is supported by emerging experience from dedicated CPIP services, including a recently described one-stop multidisciplinary groin pain pathway integrating surgical and pain management expertise [49].

### EurA-CPIP algorithm: advanced management

#### Step 6a: Surgical management

Surgical treatment for CPIP is reserved for carefully selected patients after failure of conservative and non-surgical interventions and who have undergone MDT review. Available evidence suggests that approximately 30% of patients do not achieve meaningful pain relief after CPIP surgery, and a small proportion may experience pain exacerbation or other complications [1]. Consequently, surgery for CPIP should only be considered at least 6 months after the index hernia repair, allowing adequate time for non-surgical treatments to be explored and to allow the mesh- or surgery-related inflammatory responses to decrease.

Surgical strategy is individualised and at the current evidence level, may include exploration, selective or triple neurectomy, mesh removal, or combinations thereof [50]. In addition, spermatic cord denervation can be performed as a potential add-on therapy in a trial setting [1, 51]. The choice of approach (open, laparoscopic, robotic or hybrid) depends on the primary hernia repair technique, pain distribution, physical findings, imaging when indicated, and surgeon expertise. Given the technical complexity and risk profile, CPIP surgery should be performed in specialised centres by surgeons with substantial experience in remedial groin surgeries, as recommended in the HerniaSurge guidelines and recent evidence [1, 50].

##### Mesh removal

Mesh-related pain may involve nociceptive inflammatory mechanisms, neuropathic nerve involvement, mechanical factors or a combination of these. Differentiation between these mechanisms is often difficult, and clear structural mesh abnormalities are not consistently identified. A discrete meshoma, mesh folding or mesh-related mass effect should primarily be considered when supported by the clinical presentation, physical findings or targeted imaging [52–54].

The decision to perform partial or complete mesh removal is based on intraoperative findings and MDT consensus. Reported success rates for mesh removal range from 60% to 100%, although outcomes are frequently confounded by concomitant neurectomy [1, 52, 53, 55]. Pure mesh removal without nerve resection has not been adequately studied to date and therefore cannot be supported. It is also important to note that laparoscopic (or robot-assisted) mesh removal (after preperitoneal mesh implantation) is a much more complex procedure than mesh removal after Lichtenstein repair, with potential serious complications including major vascular injury during preperitoneal dissection [1, 52, 53, 55, 56]. Thus, surgical exploration should not be undertaken solely in the expectation of identifying a correctable mesh abnormality. The indication for mesh removal or combined remedial surgery should be based on a clinical hypothesis based on pain distribution and triggers, physical examination, primary repair technique and imaging, previous treatment responses and MDT assessment.

Mesh removal carries a risk of hernia recurrence, although the reported recurrence rates are relatively low [52]. In the EurA-CPIP framework, a concurrent tissue repair is recommended (Shouldice repair preferably if feasible), but the final reconstructive strategy is left to the discretion of the operating hernia surgeon.

##### Neurectomy

Surgical neurectomy is primarily considered for neuropathic CPIP after hernia repair, particularly when symptoms and examination suggest involvement of the IIN/IHN and/or GBGFN distribution and conservative measures have failed. Contemporary guidance recognises neurectomy as a potential option in carefully selected patients but emphasises that the evidence base is heterogeneous [1, 57].

Both open selective neurectomy (tailored to the clinically implicated nerve(s)) and posterior/retroperitoneal laparoscopic neurectomy have been reported as feasible in observational series, including matched/controlled cohort data for a transabdominal retroperitoneal approach [58]. The best comparative evidence is the GroinPain Trial [30], a randomised study in patients with chronic neuropathic pain after primary Lichtenstein repair who achieved >50% pain reduction after a diagnostic tender-point infiltration. At 6 months, selective neurectomy achieved treatment success (defined as >50% VAS pain reduction) in 71% of patients (including cross-over patients 65%) [30]. From a pathophysiological perspective, proximal resection of the pain-generating nerve is plausible; however, current data are insufficient to favour selective over triple neurectomy, and comparative evidence between open versus laparoscopic approaches remains limited. Furthermore, the more proximal resection of a nerve has the potential of irreversible damage to the motor function of the nerve, leading to bulging of the lateral abdominal wall muscles. Therefore, the choice of technique should be individualised based on pain distribution, prior repair (anterior vs. posterior), suspected nerve involvement, and intraoperative findings, ideally within a multidisciplinary framework and with systematic outcome registration [1].

A pure retroperitoneoscopic approach permits proximal identification and division of the ilioinguinal, iliohypogastric and genitofemoral nerves outside the previously operated groin and preperitoneal fields. This may facilitate nerve identification while avoiding dissection within scarred operative planes. A recent technical publication has described retroperitoneoscopic triple neurectomy for postoperative chronic inguinal neuropathic pain [59]. However, comparative evidence remains limited, and current data are insufficient to establish the superiority of the pure retroperitoneal approach over open, transabdominal or other techniques. The approach should therefore be restricted to carefully selected patients and performed by surgeons with relevant specialist expertise.

#### Step 6b: Advanced systemic analgesic therapy

In patients with predominantly neuropathic or nociplastic CPIP in whom surgery is not indicated, is not desired, or has failed, escalation of pharmacological treatment may be considered under specialist supervision. This approach should be regarded as symptom-modifying rather than curative, and treatment should be individualised, time-limited where possible, and regularly reviewed. Long-term use of opioids is generally discouraged. First-line agents include tricyclic antidepressants, serotonin–noradrenaline reuptake inhibitors (preferably duloxetine), and gabapentinoids, which demonstrate moderate efficacy in neuropathic pain syndromes. Combination therapy (e.g., gabapentin with nortriptyline) may reduce dose-dependent adverse effects, although evidence for true synergistic benefit is limited. Treatment should be individualised and regularly reviewed [27].

For refractory cases, short-term intravenous therapies may be considered in specialised centres. Intravenous lidocaine infusions (3–7.5 mg/kg) may provide transient pain relief lasting less than 4 weeks in selected patients but require cardiac monitoring [60, 61]. Intravenous ketamine (0.25–1 mg/kg/day) may be considered for short-term analgesia in severe refractory cases, with careful monitoring for neuropsychiatric and systemic adverse effects [60]. These interventions are regarded as rescue therapies rather than long-term solutions and require MDT approval.

#### Step 7: Invasive non-surgical interventions

##### Neuromodulation

Neuromodulation, particularly dorsal root ganglion stimulation (DRG-S), has been explored for refractory neuropathic groin pain phenotypes compatible with CPIP. DRG-S is conceptually attractive because it can target focal dermatomal pain distributions in the groin region more precisely than conventional spinal cord stimulation [11]. However, the CPIP-specific evidence remains limited and is largely based on small case series and retrospective reports; a 2019 review of DRG neuromodulation for chronic postsurgical pain identified only small post-herniorrhaphy subgroups, with incomplete and heterogeneous reporting of outcomes and adverse events [62]. Consistent with this, the HerniaSurge guideline update notes that early findings suggest DRG neuromodulation may be effective for chronic neuropathic groin pain, but overall evidence quality is low and robust comparative data are lacking [1]. Neuromodulation may thus be considered an exceptional investigational option in highly selected refractory cases after MDT review and referral to a specialised neuromodulation centre.

##### Epidural block

Epidural block is not an evidence-based standard treatment for CPIP, but may be considered in exceptional, highly selected refractory cases as an exploratory diagnostic or therapeutic intervention after MDT review. Although robust evidence is lacking, it may provide exploratory information regarding the relative contribution of peripheral pain mechanisms. Its role remains uncertain, and all cases should be documented systematically.

#### Step 1–7: Supportive and rehabilitative care across the EurA-CPIP pathway

For patients with persistent CPIP despite adequate pharmacological and interventional management, supportive and rehabilitative care should be offered with the primary aim of functional restoration, improved self-management, and sustained participation in everyday life. Psychological and behavioural factors (including anxiety, depressive symptoms, and pain catastrophising) can meaningfully influence pain-related disability and coping and should therefore be addressed as part of comprehensive CPIP care. Cognitive behavioural therapy (CBT) has the most consistent evidence base among psychological interventions for chronic pain. A large Cochrane review concluded that CBT and related psychological therapies produce small to very small improvements in pain, disability, and distress, supporting its use when the goal is improved functioning rather than analgesia alone [63]. Acceptance and commitment therapy (ACT) may improve pain-related functioning and psychological flexibility, although effects on pain intensity are typically modest, and the certainty of evidence varies across outcomes [64]. Mindfulness-based interventions show similarly modest effects on pain with potential benefits for depression symptoms and quality of life in chronic pain populations [65]. Within EurA-CPIP, supportive and rehabilitative treatments are treated as an integral component of multidisciplinary care rather than a treatment of last resort. We therefore propose to initiate this coping trajectory as soon as possible, especially in high-risk patients (see Step 5) or patients with predominantly nociplastic pain. Outcomes related to function, work participation, and quality of life should be recorded systematically to enable benchmarking across centres and to support future refinement of the algorithm.

## Discussion

Chronic postoperative inguinal pain (CPIP) remains one of the most challenging long-term complications following inguinal hernia repair. Despite its substantial impact on quality of life and healthcare utilisation, evidence guiding optimal management is sparse, fragmented, and frequently subject to selection bias. The European Algorithm and Strategy for Management of Chronic Postoperative Inguinal Pain (EurA-CPIP) was developed to address these shortcomings by providing a pragmatic, multidisciplinary framework that integrates the best available evidence with expert consensus and prospective data collection. The parallel collection of data will allow for continuous evaluation of treatment effects at a larger scale than a specific centre can collect, and to form the network and gather patient material for any intervention trials over time.

A key limitation of previously proposed CPIP algorithms is the lack of standardisation in patient assessment, treatment sequencing, and outcome reporting [5, 9–19]. Many existing pathways are predominantly surgically oriented and insufficiently integrate pain medicine perspectives, despite the fact that patients with CPIP often present to both surgeons and pain specialists [1]. EurA-CPIP explicitly bridges this gap by embedding multidisciplinary evaluation throughout the treatment pathway and by recognising CPIP as a complex condition with nociceptive (inflammatory), neuropathic, mechanical and nociplastic components. This integrated approach reflects contemporary understanding of chronic postsurgical pain and aligns with broader pain medicine frameworks.

The step-up structure of EurA-CPIP prioritises the least invasive effective treatments, reserving surgical and advanced interventional therapies for carefully selected patients. This principle is particularly important in CPIP, where the risk of treatment-related harm, including pain exacerbation, is non-negligible [66]. Surgical series consistently report that up to 30 per cent of patients fail to achieve meaningful pain relief following remedial surgery, underscoring the need for rigorous patient selection and realistic expectation management [1]. By mandating a multidisciplinary team review before escalation to surgery or advanced interventions, the algorithm aims to minimise inappropriate procedures and optimise outcomes.

A central innovation of EurA-CPIP is its emphasis on prospective, standardised outcome measurement across participating centres. The absence of uniform outcome definitions has been a major obstacle to evidence synthesis in CPIP research. By incorporating validated instruments for pain intensity, functional impairment, psychological factors, and global improvement, the algorithm facilitates consistent evaluation of treatment effectiveness. Importantly, success is not defined solely by pain reduction but incorporates functional outcomes and patient-reported global improvement, reflecting a patient-centred perspective increasingly advocated in surgical research [67].

The integration of psychological assessment and supportive treatments further distinguishes EurA-CPIP from earlier algorithms. Psychological comorbidities, including anxiety, depression, and pain catastrophising, are common in patients with chronic postsurgical pain and are associated with poorer outcomes [68]. Routine use of validated tools such as the Hospital Anxiety and Depression Scale (HADS) and the Pain Catastrophizing Scale (PCS) enables early identification of patients at risk for suboptimal recovery [69]. Incorporating psychological interventions alongside medical and surgical treatments reinforces a biopsychosocial model of care and acknowledges that successful CPIP management extends beyond anatomical correction.

EurA-CPIP also provides a structured framework for evaluating emerging and investigational therapies. Interventions such as pulsed radiofrequency (PRF), cryoneurolysis, botulinum toxin-A (BoNT-A) injections, and neuromodulation are increasingly employed in clinical practice despite limited high-quality evidence. By embedding these treatments as predefined add-ons within a step-up algorithm and requiring systematic outcome registration, EurA-CPIP creates an infrastructure for generating real-world evidence. This approach balances innovation with patient safety and offers a pathway for refining or discontinuing therapies based on accumulated data.

The dynamic nature of the algorithm represents another important advancement. Rather than presenting a static treatment pathway, EurA-CPIP is designed to evolve through annual iterative revision informed by the prospectively collected registry data and updated literature review. This iterative process acknowledges the current limitations of the evidence base while creating a mechanism for structured evidence generation. By documenting prior treatments, phenotypes, and responses at each step, the pathway may also support better-characterised and more selectively enriched populations for future clinical studies. Finally, EurA-CPIP aligns with contemporary approaches to guideline development that emphasise adaptability and learning healthcare systems.

Beyond established preventive measures, future progress may depend on combining population-based hernia registries with detailed phenotyped CPIP cohorts. National registries [70] can provide the unselected surgical denominator, surgical characteristics and longitudinal outcomes needed to develop and validate prediction models analog to models in other surgical populations [71]. EurA-CPIP can complement these data with detailed clinical phenotyping and treatment-response information among patients who develop CPIP. Linkage within national healthcare systems, together with harmonised analyses across countries, may therefore support both population-level risk prediction and more precise characterisation of both prognosis and treatment response in CPIP. Such combined data sources could ultimately enable risk-adapted follow-up and prospective evaluation of preventive and early interventions.

Several limitations merit consideration. First, although EurA-CPIP is grounded in the best available evidence, many recommendations necessarily rely on expert consensus due to the paucity of randomised controlled trials in CPIP [1]. This limitation is explicitly acknowledged within the algorithm, and areas of uncertainty are intentionally preserved rather than masked by overly prescriptive recommendations. Second, the success of the algorithm depends on consistent implementation across centres, including adherence to outcome measurement protocols and timely data entry. Variability in local resources, reimbursement structures, and access to specialised interventions may affect generalisability. Third, while the registry-based approach enables large-scale observational analyses, causal inference remains limited, and prospective comparative studies will still be required to establish treatment efficacy.

In conclusion, the EurA-CPIP provides a structured multidisciplinary framework designed to address the heterogeneity and evidence gaps limiting previous management strategies. By integrating evidence-based principles, expert consensus, and prospective outcome registration within a dynamic framework, it provides a transparent platform for evaluating existing and emerging therapies while minimising unnecessary invasiveness and harm. Through international collaboration, the EurA-CPIP initiative aims to improve clinical decision-making by harmonising CPIP care across specialised centres and accelerate the generation of robust evidence to guide future practice.

## Data Availability

The original contributions presented in the study are included in the article/Supplementary Material, further inquiries can be directed to the corresponding author.
