# Supplementary material for: A European algorithm and strategy for management of chronic postoperative inguinal pain (EurA-CPIP)
Source: J Abdom Wall Surg. 2026 Oct 14;5:16944. doi: 10.3389/jaws.2026.16944 (PMC13713437; doi:10.3389/jaws.2026.16944)
Supplement: Supplementary file 1 [file Table1.docx]

**Appendix 1**. **Inguinal Pain Assessment Form**

**Patient ID: ……………………………………	Date:……/……/………… (DD/MM/YYYY)**

**Name: …………………………………………	M/F**

**Date of birth: ……/……/………… (DD/MM/YYYY) Doctor:…………………………**

**Hospital: ………………………...................**

Include all previous groin operation and pain treatment reports

1. Initial operation: Date: .............../................/..................	(DD/MM/YYYY)

EHS Classification: ………………………

Type of repair          □ Lichtenstein	□ Shouldice	□ Hernia sac resection

□ Plug & patch	□ TEP	□ TAPP	□ TREPP

□ TIPP	□ Other:………………………………..

Mesh type: .................................................................................................................................

Other previous surgery:	□ Pfannenstiel	□ Vasectomy	□ Open appendectomy

□ Laparoscopy	□ Other ……………………………………………………………..........

2.	Symptomatic groin:	□ right	□ left	□ both (fill out separate form for other side)

Since:…......months/years

3. Onset of pain symptoms:

□ pain persisting after hernia repair

□ immediately postoperatively	□ ………months postoperatively

4. Difference with preoperative situation:

□ new pain complaints

□ same pain as pre-operatively

intensity          □ idem             □ worse            □ better

5.	Frequency: □ rare( <1x/ week)	□ occasional(1-7x/week)	□ regular (once daily)	□ always

6.	Course of pain intensity over time (after initial operation):

□ decreasing	□ constant	□ intermittent	□ progressive

7. Localisation: □ inguinal area	□ upper leg (□ medial	□ lateral)

□ testis (orchialgia),	□ skin of scrotum, labia

□ other:……………………………………..................

Irradiation: .....................................................................................................................................

**Pain history and notes:**

…………………………………………………………………………………………………...…………………………………………………………………………………………………...…………………………………………………………………………………………...............

**Comorbidities:**

| ICD10 code | Name | Since |
| --- | --- | --- |
|  |  |  |
|  |  |  |
|  |  |  |
|  |  |  |
|  |  |  |

HAND-OUT QUESTIONNAIRE 8.-14.

8.	NRS rest (lying comfortably) 	0-10:

NRS in activity	0-10:

NRS worst (1 week) 	0-10:

NRS lowest (1 week)	0-10:

NRS average (1 week) 	0-10:

9. **DN4a**

Does the pain have one or more of the following characteristics?

Burning	□ yes	□ no

Painful cold	□ yes	□ no

Electric shocks	□ yes	□ no

Is the pain associated with one or more of the following symptoms in the same area?

Tingling	□ yes	□ no

Pins and needles	□ yes	□ no

Numbness	□ yes	□ no

Itching	□ yes	□ no

Total score :………………

10. **AAS** 1= No difficulty, 2= A little difficulty, 3= Some difficulty, 4= A lot difficulty, 5= Not able to do it, 6= did not do for other reasons

☐ Lying in bed

☐ Sitting

☐ Getting in or out bed or chair

☐ Reaching or stretching

☐ Lifting 3 to 5 pounds

☐ Walking around inside

☐ Climbing up or down stairs

☐ Walking outside or at work

☐ Engaging in sedentary activities such as typing, talking on the phone, playing cards, or watching tv

☐ Engaging in moderate physical activities, such as sweeping, washing the car, dancing, or playing golf

☐ Engaging in vigorous activities such as construction work, shovelling, playing tennis or basketball, weight lifting

☐ Engaging in sexual intercourse

Total score:………………

11. **Pain during or after ejaculation**

0= No pain at all, 1= Mild, 2= Moderate, 3= Severe, 4= Very severe

1. ☐ Intensity of pain during or after ejaculation.

0= Never any pain, 1= Pain between 0% and 50% of the time, 2= Between 50% and 75% of the time, 4= Always

2. ☐ Frequency of pain during or after ejaculation

0= No impact on my sexual activities, 2= The pain affects my sex life but does not cause me to abstain from sexual activity, 4= The pain causes me to abstain from any sexual activity

3. ☐ Impact of pain during or after ejaculation on your sexual activities

Total Score (range 0–12): ………………

12.	**PCS**

0= not at all, 1= to a slight degree, 2= to a moderate degree, 3= to a great degree, 4= all the time

*When I’m in pain*

1. ☐ I worry all the time about whether the pain will end
2. ☐ I feel I can’t go on.
3. ☐ It’s terrible and I think it’s never going to get any better.
4. ☐ It’s awful and I feel that it overwhelms me.
5. ☐ I feel I can’t stand it anymore.
6. ☐ I become afraid that the pain will get worse.
7. ☐ I keep thinking of other painful events.
8. ☐ I anxiously want the pain to go away.
9. ☐ I can’t seem to keep it out of my mind.
10. ☐ I keep thinking about how much it hurts.
11. ☐ I keep thinking about how badly I want the pain to stop.
12. ☐ There’s nothing I can do to reduce the intensity of the pain.
13. ☐ I wonder whether something serious may happen

Total score: ………………

13. **SF 12**

1= Excellent, 2= Very good, 3= Good, 4= Fair, 5= Poor

1. ☐ In general, would you say your health is

The following questions are about activities you might do during a typical day. Does your health now limit you in these activities? If so, how much?

1= Yes, limited a lot, 2= Yes, limited a little, 3= No, not limited at all

2. ☐ Moderate activities such as moving a table, pushing a vacuum cleaner, bowling, or playing golf
3. ☐ Climbing several flights of stairs

During the past 4 weeks, have you had any of the following problems with your work or other regular daily activities as a result of your physical health?

1= Yes, 2= No

4. ☐ Accomplished less than you would like
5. ☐ Did work or activities less carefully than usual

During the past 4 weeks, how much did pain interfere with your normal work (including work outside the home and housework)?

1. ☐ Not at all, 2. ☐ A little bit, 3. ☐ Moderately, 4. ☐ Quite a bit, 5. ☐ Extremely

These questions are about how you have been feeling during the past 4 weeks. For each question, please give the one answer that comes closest to the way you have been feeling. How much of the time during the past 4 weeks.

1= All of the time, 2= Most of the time, 3= A good bit of the time, 4= Some of the Time, 5= A little of the time, 6= None of the time

9. ☐ Have you felt calm & peaceful?
10. ☐ Did you have a lot of energy?
11. ☐ Have you felt downhearted and blue?

1= All of the time, 2= Most of the time, 3= Some of the time, 4= A little of the time, 5= None of the time

12. ☐ During the past 4 weeks, how much of the time has your physical health or emotional problems interfered with your social activities (like visiting friends, relatives, etc.)?

Total score: ………………

14. **HADS**

**Anxiety scale**

0= Not at all, 1=From time to time, occasionally, 2= A lot of the time, 3= Most of the time

1. ☐ I feel tense or ‘wound up’

0= Not at all, 1= Occasionally , 2= Quite often, 3= Very often

2. ☐ I get a sort of frightened feeling like ‘butterflies in the stomach’

0= Not at all, 1= A little, but it doesn’t worry me, 2= Yes, but no too badly, 3= Very definitely and quite badly

3. ☐ I get a sort of frightened feeling as if something awful is about to happen

0= Not at all, 1= Not very much, 2= Quite a lot, 3= Very much indeed

4. ☐ I feel restless as I have to be on the move

0= Only occasionally, 1= From time to time, but not too often, 2= A lot of the time, 3= A great deal of the time

5. ☐ Worrying thoughts go through my mind

0= Not at all, 1= Not very often, 2= Quite often, 3= Very often indeed

6. ☐ I get sudden feelings of panic

0= Definitely, 1= Usually, 2= Not often, 3= Not at all

7. ☐ I can sit at ease and feel relaxed

Total score (Anxiety): ………………

**Depression scale**

0= Not at all, 1= Sometimes, 2= Very often, 3= Nearly all the time

1. ☐ I feel as if I am slowed down

0= Definitely as much, 1= Not quite so much, 2= Only a little, 3= Hardly at all

2. ☐ I still enjoy the things I used to enjoy

0= I take just as much care as every, 1= I may not take quite as much care, 2= I don’t take as much care as I should, 3= Definitely

3. ☐ I have lost interest in my appearance

0= As much as I always could, 1= Not quite so much now, 2= Definitely not so much now, 3= Not at all

4. ☐ I can laugh and see the funny side of things

0= As much as I ever did, 1= Rather less than I used to, 2= Definitely less than I used to, 3= Hardly at all

5. ☐ I look forward with enjoyment to things

= Most of the time, 1= Sometimes, 2= Not often, 3= Not at all

6. I feel cheerful

0= Often, 1= Sometimes, 2= Not often, 3= Very seldom

7. I can enjoy a good book or radio or TV program

Total score (Depression): ………………

15.	Other chronic pain syndromes present:	□ no	□ yes

Please specify:………………………………………………………

**Specific questions:**

16.	Inguinal bulge present:	□ yes	□ no

17.	Pain at flexion,	□ yes	□ no

exo-,	□ yes	□ no

endorotation of the hip:	□ yes	□ no

18.	Pain related to the menstrual cycle:	□ yes	□ no

□ not applicable

Pain related to defecation	□ yes	□ no

19.	Urological complaints	□ yes	□ no

Please specify:………………………………………………………

**Current work situation:**	□ salaried employed	□ self-employed	□ unemployed

□ disabled	□ retired	□ student	□ blue-collar	□ white-collar

**Effect of pain on work status:** □ none	□ changed jobs after last operation due to pain

□ stopped working due to pain

□ works part-time due to pain

□ sick leave due to pain

**Use of analgesics	Dosage	Frequency**

Paracetamol

NSAID

Opioids

Gabapentinoids

Other……………………

**Physical examination (indicate right/ left):**

*Inspection:*

- Bulges (□ right □ left )	□ inguinal	□ femoral
- Scars (draw)

*Somatosensory disturbances: (draw)* **PINPRICK MAPPING 24 squares**

"Created in BioRender. van der Meer, T. (2026) https://BioRender.com/kk712y4 ".

“Created in BioRender. van der Meer, T. (2026) https://BioRender.com/ eex8x3b”

- Pinprick test: □ right □ left

□ normal (0) □ ane-hypoesthesia (-) □ hyperalgesia (+) □ allodynia (++)

- Vital (cold perception): □ right □ left

□ normal	□ Disturbed

- Punctum maximum trigger pain (X)

**DN4b**

Is the pain located in an area where the physical examination may reveal one or more of the following characteristics?

Hypoesthesia to touch	□ yes	□ no

Hypoesthesia to pinprick	□ yes	□ no

In the painful area, can the pain be caused or increased by:

Brushing?	□ yes	□ no

*Palpation:*

- Inguinal bulge	(□ right □ left )	□ yes	□ no
- Femoral bulge	(□ right □ left )	□ yes	□ no

Pain during funicular traction (□ right □ left )	□ yes	□ no

Pain pressing pubic tubercle (□ right □ left )	□ yes	□ no

Pain pressing adductor tendons (□ right □ left )	□ yes	□ no

Pain pressing testicle  (□ right □ left)	□ yes	□ no

Pain pressing epididymis (□ right □ left)	□ yes	□ no

Trigger point (draw with x) (□ right □ left )	□ yes	□ no

Radiation to:………………………………………………….

*Specific tests:*

- Pinch test:	□ positive	□ negative
- Laseque’s test:	□ positive	□ negative
- Adductor test:	(□ right □ left )	□ positive	□ negative
- Pain at flexion/ endo-exorotation hip: (□ right □ left )	□ positive	□ negative

**Additional imaging (date and finding):**

□ X-ray –pelvis/ hip: ……………………………………………………………………………

□ Ultrasonography	……………………………………………………………………………

□ CT-scan: ………………………………………………………………………………………

□ MRI: ………………………………………………………………………………………….

□ Bone scan: ……………………………………………………………………………………

□ Other:…………………………………………………………………………………………..

**Differential diagnosis and other consultations:**

□ **Urology**

□ **Gynaecology**

□ **Physical Medicine & Rehabilitation**

□ **Orthopaedics**

□ **Neurosurgery**

□ **Gastroenterology**

**Type infiltration (repeat max 3 times with min 2 weeks interval):**

- Trigger point
  - □ Blind	□ US guided
  - □ pubic bone	□ funicular	□ other: ………………………
- Nerve block:
  - □ Blind	□ US guided
  - □ Iliohypogastric/ilioinguinal nerve

□ Genital branch	□ LFCN

- □ other:…………..	□ TAP	□ paravertebral

□ lidocaine conc……: …..ml □ bupivacaine conc……: …..ml □

levobupivacaine: conc……: …..ml

□ ropivacaine: …..ml

□ Other………….

□ Corticosteroids:………ml (type and concentration……….)

| **Average Pain level one week before infiltration:** |  |  | | |  | |  | |  | |  | |  | |  |
| --- | --- | --- | --- | --- | --- | --- | --- | --- | --- | --- | --- | --- | --- | --- | --- |
| **NRS** (0= no pain, 10= worst pain imaginable):……  **Pain level after 5-10 minutes after infiltration:**  **NRS** (0= no pain, 10= worst pain imaginable):……  **PGI-I What describes how your post-infiltrative condition is now compared with how it was before infiltration?** (1= Very much better, 2= Much better, 3= A little better, 4= No change, 5= A little worse, 6= Much worse, 7= Very much worse) …….  **Average pain level 2 weeks after infiltration:**  **NRS** (0= no pain, 10= worst pain imaginable):……  **PGI-I What describes how your post-infiltrative condition is now compared with how it was before infiltration?** (1= Very much better, 2= Much better, 3= A little better, 4= No change, 5= A little worse, 6= Much worse, 7= Very much worse)……. | | |  |  | |  | |  | |  | |  | |  | |

**Treatment:**

□ Expective

□ Medication, specify……………………………………………………………………….

□ Additional nerve blocks, specify……………………………………………………….....

□ Cryoneurolysis, specify…………………………………………………………………….

□ Pulsed Radiofrequency Ablation (PRF) specify…………………………………………

□ Recurrent inguinal hernia repair

□ Mesh removal (□ open	□ laparoscopic	robotic )

□ Neurectomy	(□ Iliohypogastric	□ Ilioinguinal	□ Genital branch

□ open □ laparoscopic	robotic )

□ Other: …………………………………………………………………………………....
