# Supplementary material for: A European algorithm and strategy for management of chronic postoperative inguinal pain (EurA-CPIP)
Source: J Abdom Wall Surg. 2026 Oct 14;5:16944. doi: 10.3389/jaws.2026.16944 (PMC13713437; doi:10.3389/jaws.2026.16944)
Supplement: Supplementary file 2 [file Table2.docx]

| **Step** | **Ref #** | **Study (first author, year, journal)** | **Design** | **N** | **Main finding** | **Role in algorithm** |
| --- | --- | --- | --- | --- | --- | --- |
| **0 - History/exam & prevention** | 1 | Stabilini et al. 2023, *BJS Open* | Guideline | - | Updated international consensus recommendations for groin hernia/CPIP management | Underpins prevention and CPIP treatment management |
|  | 7 | Miller et al. 2023, *Hernia* | Retrospective chart review | N=61 | Psychological disorders are common among CPIP patients | Supports psychosocial screening |
|  | 8 | HerniaSurge Group 2018, *Hernia* | Guideline | - | Original international guideline for groin hernia management | Foundational guideline |
|  | 26 | Findlay et al. 2025, *Frontiers in Surgery* | Narrative review | - | Advocated prevention-focused research for CPIP | Frames prevention |
| **1 - Systemic pharmacotherapy** | 27 | Finnerup et al. 2015, *The Lancet Neurology* | SR/meta-analysis of RCTs | 229 studies (N=NR) | TCAs, SNRIs and gabapentinoids are first-line for neuropathic pain, and opioids are not first-line | Basis for Step 1 |
| **2 - Topical therapy** | 1 | Stabilini et al. 2023, *BJS Open* | Guideline | - | No benefit has been shown for capsaicin/lidocaine patch treatment, however not contraindicated due to low risk | Supports offering topical therapy despite low-evidence |
|  | 28 | Bischoff et al. 2013, *Anesthesiology* | RCT, double-blind, placebo-controlled, crossover | N=21 | Lidocaine 5% patch showed no significant benefit over placebo | Basis for stating limited evidence |
| **3 - Trigger point injections** | 1 | Stabilini et al. 2023, *BJS Open* | Guideline | - | Notes TPI is relevant after Lichtenstein repair. States no evidence-based recommendation exists for free-hand vs ultrasound-guided technique | Frames TPI as standard step |
|  | 29 | Wijayasinghe et al. 2016, *British Journal of Anaesthesia* | RCT, double-blind, placebo-controlled, crossover | N=14 (and 6 healthy volunteers) | Peripheral input from the tender-point area maintains spontaneous and evoked pain | Support a peripheral pain generator rationale for TPI |
|  | 30 | Verhagen et al. 2018, *Annals of Surgery* | RCT | N=54 | TPI success 22% vs tailored neurectomy 71% | Supports step-up TPI -> neurectomy |
| **4 - Diagnostic nerve blocks** | 31 | Bischoff et al. 2012, *Anesthesia & Analgesia* | RCT, double-blind, placebo-controlled, crossover | N=12 (+12 controls) | US-guided IIN/IHN lidocaine blocks produced transient sensory changes but were not reliably diagnostic | Evidence base for diagnostic block role |
|  | 32 | Thomassen et al. 2013, *Hernia* | Retrospective cohort | N=38 | IIN/IHN nerve blocks can be effective to treat CPIP. The use of US was not superior to nerve stimulator guided blocks | Supports use of nerve blocks, informs guidance-technique |
| **4a - Pulsed radiofrequency** | 33 | Werner et al. 2012, *Regional Anesthesia & Pain Medicine* | Systematic review | 4 studies (N=8) | Evidence for PRF in persistent pain after inguinal herniotomy is fairly limited | Basis for labelling PRF investigational |
| **4b - Cryoneurolysis** | 34 | Biel et al. 2023, *Pain practice* | Narrative review | - | Overview of cryoneurolysis mechanism and applications for acute and chronic pain | Background rationale for cryoneurolysis mechanism |
|  | 35 | Callesen et al. 1998, *Anesthesia & Analgesia* | RCT, prospective, randomised, observer- and patient-blinded | N=48 | Perioperative cryoanalgesia of the IIN/IHN during primary hernia repair did not reduce postoperative pain compared with sham treatment. | Provides negative early perioperative evidence; does not directly evaluate cryoneurolysis for established CPIP |
|  | 36 | Campos et al. 2009, *Pain Physician* | Case report | N=1 | US-guided cryoablation of FBGFN gave complete pain relief, sustained at 2-month follow-up | Supports feasibility of US-guided technique |
|  | 37 | Fanelli et al. 2003, *Surgical Endoscopy And Other Interventional Techniques* | Case series | N=10 (12 procedures) | Cryoanalgesic ablation was associated with substantial pain reduction and reduced analgesic use in a small uncontrolled series | Supports feasibility in postherniorrhaphy neuropathic patients |
|  | 38 | Ilfeld et al. 2017, *British Journal of Anaesthesia* | Narrative review | - | Proposes percutaneous cryoneurolysis as an alternative to peripheral nerve blocks | Supports renewed interest in US-guided cryo |
|  | 39 | Ilfeld et al, 2020, *Anesthesiology* | Narrative review | - | Reviews cryoneurolysis/percutaneous PNS vs opioids and LA techniques | Basis for investigational status |
| **4c - Botulinum toxin A** | 40 | Ranoux et al. 2008, *Annals of Neurology* | RCT | N=29 | BoNT-A produced direct analgesic effect in chronic neuropathic pain | Supports antinociceptive mechanism |
|  | 41 | Hary et al. 2022, *European Journal of Pain* | SR/meta-analysis | 10 studies (N=505) | BoNT-A effective and safe across chronic peripheral neuropathic pain syndromes | Supports general efficacy and safety rationale |
|  | 42 | Attal et al. 2016, The Lancet Neurology | RCT, multicentre, double-blind, placebo-controlled | N=66 | Repeated BoNT-A injections safe and effective for peripheral neuropathic pain | Supports repeat dosing safety and efficacy |
|  | 43 | Aoki et al, 2011, *Parkinsonism & Related Disorders* | Narrative review | - | BoNT-A inhibits release of pain-related neuropeptides from peripheral sensory terminals and may attenuate peripheral sensitisation; central effects remain uncertain | Mechanistic rationale for BoNT-A use |
|  | 44 | Oh et al. 2015, *Toxins* | Narrative review | - | Summarises mechanism of action and evidence for BoNT-A in neuropathic pain | Supplementary evidence |
|  | 45 | Egeo et al. 2020, *Frontiers in Neurology* | Narrative Review | - | BoNT-A mechanisms and evidence base in neuropathic pain | Supplementary evidence |
|  | 46 | Wang et al. 2024, *Frontiers in Neurology* | SR/meta-analysis of RCTs | 14 studies (N=1358) | BTX-A gave lower pain scores and higher effective rate vs lidocaine/gabapentin, with no increase in adverse effects | Neuropathic syndrome evidence (postherpetic neuralgia) |
|  | 47 | Tereshko et al. 2023, *Frontiers in Neurology* | Case report | N=1 | BoNT-A improved symptoms in genitofemoral neuralgia | Direct CPIP-adjacent case |
|  | 48 | Calixte et al. 2017, *Current urology reports* | Narrative review | - | Treatment pathway for chronic testicular/groin pain, including BoNT-A | Supports BoNT-A as option |
| **5 - MDT review** | 23 | Bjelland et al. 2002, *Journal of Psychosomatic Research* | Narrative review | - | Validated HADS cutoffs (≥8 possible ≥11 probable anxiety/depression) | Supports the HADS screening thresholds used to identify patients who may benefit from early multidisciplinary assessment |
|  | 24 | Sullivan et al. 1995, *Psychological Assessment* | Validation study | 4 studies (N=425+30+28+NR) | Developed and validated Pain Catastrophizing Scale | Basis for MDT |
|  | 49 | Cocciolo. 2025, *Journal of Abdominal Wall Surgery* | Retrospective cohort | N=40 | The one-stop multidisciplinary clinic significantly improved pain function, and satisfaction | Supports MDT structure |
| **6a - Surgery (general)** | 1 | Stabilini et al. 2023, *BJS Open* | Guideline | - | States ~30% of CPIP surgery patients fail to achieve meaningful relief | Basis for caution |
|  | 50 | Huy et al. 2025, *JAMA Surgery* | Case series, single-institution, retrospective | N=818 | Tailored surgical approaches, neurectomy, mesh excision, recurrent hernia repair; can be safe and effective in experienced centres | Supports individualised surgical strategy |
|  | 51 | Weitzner et al. 2025, *Surgical Endoscopy* | Retrospective cohort | N=173 | Patient tailored surgery improved orchialgia | Supports spermatic cord denervation as add-on therapy |
| **6a - Mesh removal** | 52 | Chaoui et al. 2025, *Surgical Endoscopy* | Prospective cohort | N=83 | Hernia recurrence occurred in approximately one in five patients after laparoscopic mesh removal, and approximately one in ten required remedial surgery | Quantifies recurrence risk following laparoscopic mesh removal |
|  | 53 | Slooter et al. 2023, *Hernia* | retrospective cohort | N=44 | Laparoscopic mesh removal after endoscopic repair reduced pain in most patients | Supports mesh removal efficacy |
|  | 54 | Zwaans et al. 2017, *World Journal of Surgery* | Retrospective cohort | N=74 | Mesh removal either or not combined with tailored neurectomy improved pain after Lichtenstein repair | Supports combined mesh removal |
|  | 1 | Stabilini et al. 2023, *BJS Open* | Guideline | - | Reports mesh removal success rates, though often confounded by neurectomy | Basis for stated success ranges |
|  | 55 | Özveri et al. 2025, *Hernia* | Retrospective cohort | N=32 | Laparoscopic mesh removal is associated with good outcomes | Supports mesh removal efficacy |
|  | 56 | Lu et al. 2021, *Surgical Technology International* | Retrospective cohort | N=196 | Vascular injury during mesh explanation should be anticipated but can be safely controlled | Basis for complexity and risk of laparoscopic mesh removal |
| **6a - Neurectomy** | 1 | Stabilini et al. 2023, *BJS Open* | Guideline | - | Recognises neurectomy as an option, notes heterogeneous evidence | Basis for neurectomy evidence |
|  | 57 | Chen et al, 2013, *JAMA Surgery* | Prospective cohort | N= 20 | Laparoscopic retroperitoneal neurectomy feasible for chronic inguinal neuralgia | Supports laparoscopic retroperitoneal approach |
|  | 58 | Karampinis et al. 2017, *BMC Surgery* | Retrospective matched -pair cohort | N=9 (+9 controls) | Provides data supporting transabdominal laparoscopic retroperitoneal neurectomy | Supports transabdominal approach |
|  | 30 | Verhagen et al. 2018, *Annals of Surgery* | RCT | N=54 | Selective neurectomy success 71% | Best comparative evidence for neurectomy |
|  | 59 | Naguib et al. 2025, Journal of Visceral Surgery | Technical note | - | Describes retroperitoneoscopic triple neurectomy technique, no comparative evidence | Introduces new technique |
| **6b** **- Advanced pharmacotherapy** | 27 | Finnerup et al. 2015, *The Lancet Neurology* | SR/meta-analysis of RCTs | 229 studies (N=NR) | see Step 1 | - |
|  | 60 | Moisset et al. 2020, *Revue Neurologique* | Systematic review + expert recommendations |  | Provides expert recommendations on intravenous lidocaine and ketamine as specialist options for selected patients with refractory neuropathic pain | Basis for IV lidocaine and ketamine |
|  | 61 | Zhu et al. 2019, *Frontiers in Neurology* | SR/meta-analysis | 26 studies (N=NR) | IV lidocaine provided transient relief in neuropathic pain | Supports IV lidocaine |
| **7 - Neuromodulation** | 11 | Liem et al. 2016, *Pain Practice* | Narrative review | - | Proposes DRG stimulation for postherniorrhaphy chronic neuropathic groin pain | Conceptual rationale for DRG in CPIP |
|  | 62 | Antony et al. 2019, *Pain medicine* | Narrative review | NR | Evidence for strong pain relief with DRG stimulation | Basis for low evidence |
|  | 1 | Stabilini et al. 2023, *BJS Open* | Guideline | - | DRG neuromodulation may be effective, but overall evidence quality is low | Frames neuromodulation as low-evidence |
| **1-7 - Supportive/rehabilitative care** | 63 | Williams et al. 2020, Cochrane Library | Cochrane SR/meta-analysis of RCTs | 75 studies (N=9401) | CBT/psychological therapies give small to very small improvements in pain, disability, and distress | Evidence basis for CBT |
|  | 64 | Hughes et al. 2017, The Clinical Journal of Pain | SR/meta-analysis | 11 studies (N=NR) | ACT improves pain-related function/psychological flexibility, modest effect on pain intensity | Evidence basis for ACT |
|  | 65 | Hilton et al. 2017, *Annals of Behavioral Medicine* | SR/meta-analysis | 38 studies (N=NR) | Mindfulness interventions show modest pain effects | Evidence basis for mindfulness |

CPIP= chronic postoperative inguinal pain; TPI= trigger point injection; IIN= ilioinguinal nerve; IHN= iliohypogastric nerve; GFN= genitofemoral nerve; FBGFN= femoral branch of genitofemoral nerve; BoNT-A= botulinum toxin type A; PRF= pulsed radiofrequency; DRG= dorsal root ganglion; MDT= multidisciplinary team; HADS= Hospital Anxiety and Depression Scale; PCS= Pain Catastrophizing Scale; RCT= randomised controlled trial; SR= systematic review; CBT= cognitive behavioural therapy; ACT= acceptance and commitment therapy; PNS= peripheral nerve stimulation; LA= local anaesthetic; IV=intravenous; US= ultrasound; NR= not reported; *k*= number of included studies
